# Incorporating Information on Control Diseases Across Space and Time to Improve Estimation of the Population-level Impact of Vaccines

**DOI:** 10.1097/EDE.0000000000001341

**Published:** 2021-03-12

**Authors:** Kayoko Shioda, Jiachen Cai, Joshua L. Warren, Daniel M. Weinberger

**Affiliations:** From the a Department of Epidemiology of Microbial Diseases, Yale School of Public Health, New Haven, CT; b Department of Biostatistics, Yale School of Public Health, New Haven, CT.

**Keywords:** Brazil, Distributed lag model, Horseshoe prior, Pneumococcal conjugate vaccine, Synthetic control model, Vaccine evaluation

## Abstract

The synthetic control method evaluates the impact of vaccines while adjusting for a set of control time series representing diseases that are unaffected by the vaccine. However, noise in control time series, particularly in areas with small counts, can obscure the association with the outcome, preventing proper adjustments. To overcome this issue, we investigated the use of temporal and spatial aggregation methods to smooth the controls and allow for adjustment of underlying trends.

**Methods:**

We evaluated the impact of pneumococcal conjugate vaccine on all-cause pneumonia hospitalizations among adults ≥80 years of age in 25 states in Brazil from 2005 to 2015. Pneumonia hospitalizations in this group indicated a strong increasing secular trend over time that may influence estimation of the vaccine impact. First, we aggregated control time series separately by time or space before incorporation into the synthetic control model. Next, we developed distributed lags models (DLMs) to automatically determine what level of aggregation was most appropriate for each control.

**Results:**

The aggregation of control time series enabled the synthetic control model to identify stronger associations between outcome and controls. As a result, the aggregation models and DLMs succeeded in adjusting for long-term trends even in smaller states with sparse data, leading to more reliable estimates of vaccine impact.

**Conclusions:**

When synthetic control struggles to identify important prevaccine associations due to noise in control time series, users can aggregate controls over time or space to generate more robust estimates of the vaccine impact. DLMs automate this process without requiring prespecification of the aggregation level.

## Abstract 2

Supplemental Digital Content is available in the text.

Vaccines are introduced for routine use in immunization programs after a careful assessment of their efficacy and safety in clinical trials. Following the widespread introduction of the vaccine, the population-level impact – a composite of direct and indirect effects and vaccine uptake – should be estimated to help decision makers and donors evaluate immunization policies.^1,2^ Administrative and surveillance databases are often the only sources of data for vaccine evaluation. Compared to other types of data (e.g., those from case control studies and cross-sectional studies), these databases generally cover larger geographic areas and a longer duration of time in both prevaccine and postvaccine periods, which are critical for vaccine evaluation. These data, however, are likely influenced by various temporal factors, such as changes in healthcare systems and surveillance effort. Unfortunately, these changes are often difficult to quantify and, therefore, are challenging to adjust for using traditional methods such as a simple pre–post comparison or trend adjustment.

The synthetic control method has been used for the evaluation of public health interventions. This method can adjust for underlying trends in an outcome of interest using information on time-varying controls that are not influenced by the interventions.^3,4^ For example, previous studies have evaluated the impact of pneumococcal conjugate vaccines (PCVs) on pneumonia hospitalizations using data on other causes of hospitalizations as controls (e.g., diseases of the circulatory system, digestive system, etc.).^5–7^ Given a large set of control disease time series, the synthetic control approach uses Bayesian variable selection techniques to select the optimal set of controls based on relationships between the outcome and controls in the prevaccine period. These estimated associations are used for predicting the counterfactual outcome in the absence of vaccines in the postvaccine period and the predictions are used to evaluate the vaccine impact. A limitation is that estimating important associations can be difficult when the control disease counts are small because of the noise in the time series.^6^ As a result, the model cannot detect and adjust for underlying trends in the prevaccine period and fails to generate accurate estimates of the counterfactuals in the postvaccine period, which are needed to describe vaccine impact.

One way to overcome the issue of control disease data sparsity is to create a “consensus trend” from smoothed control disease time series using principal component analysis, and use it in a regression analysis to control for underlying trends.^6^ A simulation study demonstrated that this approach leads to an improved estimation of vaccine impact. However, using principal component analysis results is a less interpretable solution and results in a potential loss of predictive information when compared to using the individual control disease time series for modeling and prediction. Therefore, in this study, we investigate an alternative solution to the problems caused by sparsity of control disease by incorporating additional information from neighboring geographic areas or nearby time periods into the synthetic control model to stabilize estimation of key associations. First, we use control disease time series aggregated at different levels of time (month, quarter, semester, or year) or space (state, region, or nation) in the synthetic control model, and evaluate if this approach improves the ability of the synthetic control model to estimate associations, leading to improved vaccine impact estimates. Because the most appropriate level of aggregation may be unknown a priori or may change across different control diseases, we next propose temporal and spatial distributed lag synthetic control models (DLMs), which automates the entire process. The models simultaneously select the appropriate level of aggregation separately for each control disease variable and estimate their association with the primary outcome. Another strength of the DLM is that it closely resembles the usual synthetic control model in the case where incorporating outside information is not needed to estimate associations with control diseases, suggesting that it may be used whenever auxiliary information is readily available but the user is unsure of its utility.

We apply these methods to evaluate the population-level impact of the introduction of 10-valent PCV (PCV10) in children in Brazil against all-cause pneumonia hospitalizations among adults ≥80 years old. We chose this population as an example, as Brazil has a sufficient amount of pre- and post-PCV data with a high geographic resolution. This enabled us to perform both temporal and spatial aggregation of control disease time series. We selected the elderly, which was not the age group targeted by PCV10, because previous studies identified a strong long-term increasing trend in pneumonia hospitalizations among this age group that could not be solely explained by the increasing population size.^6^ The synthetic control model was unable to adjust for this increasing trend at the state level in small states due to noise in the control disease time series data. As a result, it appeared that PCV10 had a negative impact (i.e., disease rates were higher than expected after vaccine introduction). Therefore, in this article, we aim to show that these aggregation methods can capture and control for this strong secular trend in the elderly at the state level without resorting to dimension reduction techniques.

## METHODS

### Data

We used publicly available hospital discharge data between January 2005 and December 2015 from Brazil provided by the Department of Vital Statistics, a branch of the Brazilian Ministry of Health. As Brazil introduced PCV10 in March 2010, there were 5 years and 2 months of data in the prevaccine period and 5 years and 10 months of data in the postvaccine period. Brazil has 27 states that belong to the following five regions: North (seven states), Northeast (nine states), Southeast (four states), South (three states), and Center-West (four states). We excluded two states in the North region from the analysis as their data for the elderly were unavailable. The Human Investigation Committee at Yale School of Medicine determined that this research is exempt from review.

Details of the hospitalization data can be found elsewhere.^8^ Briefly, causes of hospitalizations were recorded using International Statistical Classification of Diseases and Related Health Problems tenth revision (ICD-10) codes. The primary outcome was all-cause pneumonia hospitalizations, defined as having an ICD-10 code in the range of J12–J18. Control diseases were other ICD-10 chapters and sub-chapters (eTable 1; http://links.lww.com/EDE/B785) and were excluded from analysis if they were likely affected by PCV10 or if their relationships with the outcome changed over time.

One of the common issues with administrative data is inconsistency in the reporting system over time. As long as the inconsistency affects both the outcome and control diseases similarly, the synthetic control model should be able to generate reliable counterfactual adjusted for underlying trends. However, if changes in the reporting system affect each disease differently, that should be adjusted in order to obtain valid results from the synthetic control approach. In Brazil, the ICD-10 coding practice changed in 2008 due to the change in the government’s reimbursement policy. Before 2008, hospitals were required to submit cause-specific ICD-10 codes (e.g., J13: pneumonia due to *Streptococcus pneumoniae*) to receive reimbursement for their healthcare cost. The reimbursement scheme changed in January 2008, resulting in the increased proportion of unspecified codes (e.g., J18: pneumonia, unspecified organism).^1^ We adjusted for this shift using a cubic spline as was done previously.^5^

### Approach 1: Synthetic Control Models with Control Disease Time Series Aggregated by Time or Space

To determine if aggregating control disease data improved the performance of the synthetic control model, we investigated the following six combinations of aggregation levels for control diseases: (1) month and state-level (“reference” model without aggregation); (2) quarter and state-level; (3) semiannual and state-level; (4) annual and state-level; (5) month and regional; and (6) month and national time series (eTable 2; http://links.lww.com/EDE/B785). For each combination, every control disease variable was aggregated in the same way (i.e., no mixing of aggregation types across the set of variables). To create quarterly, semiannual, and annual control time series for a selected control disease, we calculated the mean number of control cases per respective time period (eFigure 1; http://links.lww.com/EDE/B785). Regional and national control time series are simply the number of each control disease in each region where the selected state is located or at the national level. The outcome variable (all-cause pneumonia hospitalizations) was always modeled on the monthly time scale at the state level, with individual control diseases input to the synthetic control model at the different levels of aggregation.

Given a selected combination of aggregation levels for the control disease variables, we used a modified version of the synthetic control model to relate the observed number of all-cause pneumonia hospitalizations at month *t* in a selected state (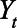) as a function of *p* different time-varying control diseases (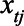, *j* = 1, …, *p*) and other nondisease related covariates, also possibly time-varying (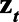). Specifically, the Poisson regression model is given as


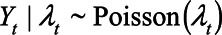


where


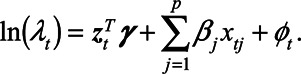


The 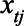 variable represents the aggregated (over space or time) value of control disease *j* corresponding to calendar month *t*. For example, for the monthly/state control disease analyses (reference model), this simply represents the number of cases of disease *j* during calendar month *t* in the selected state; for the quarterly/state analyses, this represents the average number of hospitalization in the quarter containing calendar month *t* across the state; and for monthly/regional analyses, this represents the total number of hospitalizations across the region containing the selected state in the given calendar month. Other combinations are similarly defined. We added 0.50 to each raw monthly control disease case count, log-transformed it, and adjusted it for the change in coding practice in 2008 before any aggregation taking place. The aggregated predictor was then standardized before analysis to improve the computational stability of the algorithm. The 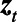 vector includes an intercept, monthly dummy variable (December serving as the reference), and indicator for the 2009 influenza outbreak (equal to 1 for August and September 2009 and 0 for all other months). We included independent, normally distributed random effects (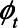), centered at zero with a shared variance parameter at each month of analysis, to account for potential overdispersion.

We performed variable selection for the control diseases by assigning the horseshoe prior distribution to the corresponding regression parameters such that 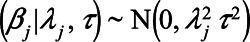 with 
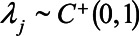
 and 
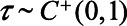
, where 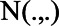 represents the normal distribution and 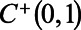 is a half-Cauchy distribution.^9,10^ For the horseshoe prior, local shrinkage of a specific regression parameter is controlled by 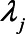, whereas 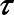 describes global shrinkage across all parameters; values close to zero for either lead to small values of the corresponding regression parameter(s) and suggest that the control disease(s) are not associated with pre-vaccine variability in pneumonia hospitalizations. A relative importance weight for control disease *j* is estimated using the posterior mean of 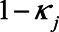, where 
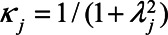
.^9,10^ An estimated weight near one indicates that the control disease had a strong relationship with the outcome in the prevaccine period. We considered a control disease as “selected” in the model if this estimated weight was >0.50. We decided to use the horseshoe prior distribution instead of the spike and slab prior distribution that was used in the previous studies because it generally led to improved model convergence.^5,6^

We fit models with control time series aggregated at the aforementioned six levels separately for each state. Models were fit to the pre-vaccine data, and the counterfactual number of pneumonia hospitalizations in the postvaccine period was generated from their posterior predictive distributions using the observed post-vaccine data on control diseases. To complete the model specification, we selected independent normally distributed prior distributions centered at zero with a standard deviation of 100 for the 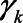 parameters, and used a uniform prior distribution for the standard deviation parameter for 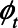
with a lower bound of zero and upper bound of 1,000. We fit the models in the Bayesian setting using JAGS.^11^ For each model, we collected 20,000 nearly independent samples from the joint posterior distribution after removing at least 200,000 iterations before the model converged (amount varied by state/level of aggregation) and thinning the total number of collected samples from two separate chains by a factor of 40 (i.e., resulting in near independence of the samples). Model convergence was evaluated based on visual inspection of trace plots and calculation of Geweke diagnostic for the posterior predicted samples of the calculated rate ratios (RRs) (see “Evaluation of the Impact of 10-valent Pneumococcal Conjugate Vaccine” section for more information on this quantity).^12^

### Approach 2: Distributed Lag Synthetic Control Models

The structure of the DLMs is similar to the previously described model. However, a key difference is that the user is not required to select a level of data aggregation a priori. Instead, the DLMs include parameters that weight each control disease covariate at different spatial or temporal “lags” based on their association with the outcome. Importantly, these weights are estimated by the prevaccine data and can differ by control disease. The key assumption is that control disease counts from states and time periods further away from the current state/time under analysis will receive less weight. Specifically, we utilize modeling techniques from and define the spatial DLM as


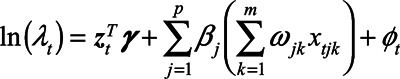


where many of the terms have been previously described and 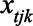 is now the count of control disease *j* during month *t* from state *k*.^13–15^ The weight corresponding to counts of control disease *j* from state *k* is denoted by 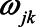 and is defined as


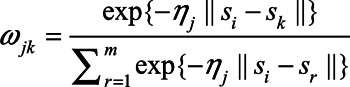


where 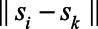 is the Euclidean distance between the centroids of states *i* and *k*, *m* is the total number of states in the analysis (i.e., 25), state 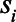 is where pneumonia hospitalization data are being analyzed from, and 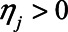 is the parameter that describes how much weight control disease *j* receives at different distances. This definition ensures that the weights sum to one, leading to a weighted average of counts from surrounding states at each time period and for each different control disease being used in the regression model. Additionally, by incorporating distances between the states, the definition ensures that states far away from the state of analysis receive smaller values. When 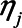 is large, this suggests that only the home spatial location receives a large weight, resulting in a model very similar to the previously described reference model. As 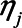 decreases, the weights at larger spatial distances increase. Allowing 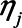 to differ by control disease means that different controls can receive different weights at the same spatial distances.

In a similar way, we also introduce the temporal DLM such as


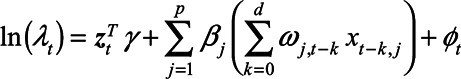


where 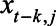 is now the count of control disease *j* from time period *t-k* and the weights are defined as


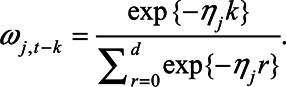


In the temporal DLM, instead of including control diseases from states surrounding the state under analysis, we include control disease counts from the state under analysis from up to *d* lags in the past. This definition ensures that counts further in the past receive less weight and again, 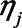 is the control disease-specific.

We fit the DLMs using JAGS with many of the prior distributions previously described. We specify a uniform prior distribution for the 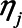 parameters with a lower bound of zero and upper bound determined by the observed spatial distances and selected maximum time lag. Convergence was monitored as previously described and we again collected a total of 20,000 approximately independent samples from the joint posterior distribution across all models.

### Evaluation of the Impact of 10-valent Pneumococcal Conjugate Vaccine

For each fitted model, we quantified the impact of PCV10 using an RR, which was defined as the cumulative number of observed J12–18 hospitalizations divided by the cumulative number of counterfactual predicted J12–18 hospitalizations (sampled from the posterior predictive distribution) in the evaluation period (from March 2011, a year after the introduction of PCV10, to December 2015). For each set of disease case predictions generated during this time period, we calculated the RR, giving us a total of 20,000 posterior predicted samples of this quantity. To summarize the RR, posterior medians were used as point estimates and the 95% highest density credible intervals (CrIs) were calculated.

### Model Comparison

We compared the performance of the models using the evidence ratio of deviance information criterion (DIC) weights,^16,17^ which was calculated as


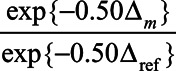


where 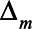 and 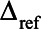
are the differences in the DIC values between the best model (i.e., the model with the smallest value of DIC) and model *m* and the reference model, respectively. The evidence ratio tells us how likely model *m* is to be the best model in comparison to the reference model. To fairly compare the DLMs to the aggregated versions, we fit each model to the exact same dataset with appropriate lag periods removed.

## RESULTS

### Approach 1: Synthetic Control Models with Control Disease Time Series Aggregated by Time or Space

**FIGURE 1.**
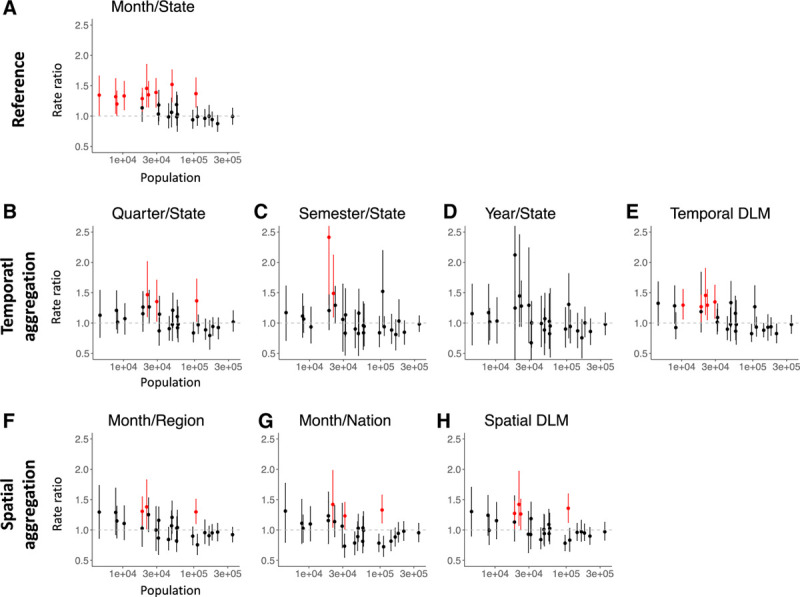
Rate ratios for adults ≥80 years of age in 25 states in Brazil by population size, estimated by the reference model (A), synthetic control models using aggregated controls (B, C, D, F, and G), and distributed lag models (E and H). Rate ratios were the cumulative number of observed all-cause pneumonia hospitalizations (ICD-10 code: J12-18) divided by the cumulative number of counterfactual pneumonia hospitalizations in the evaluation period (March 2011–December 2015). Dots and bars represent posterior medians and 95% highest density CrIs, respectively. Dots and bars are in red when rate ratios were significantly greater than one. C and D, 95% credible intervals exceeded the limit of *y* axis in a few states. Figures S2 and S3 show the full length of 95% CrIs in all states.

We estimated RRs for adults aged ≥80 years in 25 states with six different synthetic control models using either original or aggregated control time series (eTable 2; http://links.lww.com/EDE/B785). In 10 (40%) of 25 states (Rondônia, Acre, Tocantins, Pernambuco, Alagoas, Sergipe, Mato Grosso do Sul, Mato Grosso, Goiás, and Distrito Federal), the reference model generated RRs with 95% CrI lower bounds >1, suggesting that pneumonia hospitalizations increased after vaccine introduction (Figure 1A). Except for Pernambuco and Goiás, these states had small population size with the average population of adults ≥80 years of age during the study period under 30,000. Due to the data sparsity, control diseases had noise in their time series which obscured the association with the outcome, preventing the synthetic control model from identifying an appropriate set of control disease and properly adjusting for long-term increasing trends.

**FIGURE 2.**
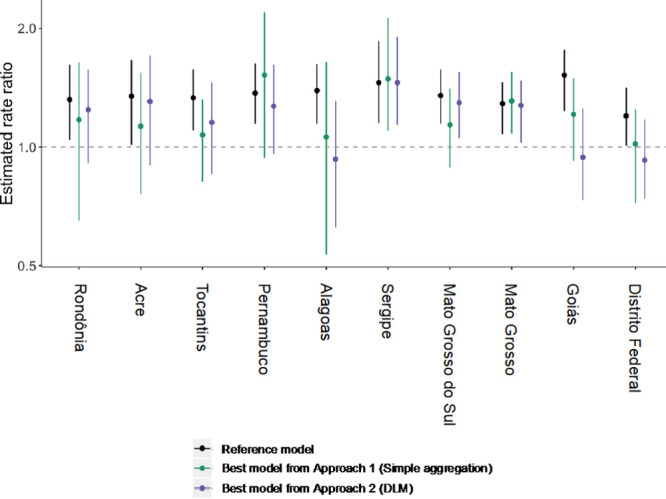
Rate ratios for adults ≥80 years of age, estimated by the reference model and the best models from approaches 1 and 2, in 10 states where the reference model generated rate ratios with 95% CrIs greater than 1. Rate ratios were the cumulative number of observed all-cause pneumonia hospitalizations (ICD-10 code: J12–18) divided by the cumulative number of counterfactual pneumonia hospitalizations in the evaluation period (March 2011–December 2015). Dots and bars represent posterior medians and 95% highest density CrIs, respectively. Best models were defined as the models with the smallest DIC values for each state.

In contrast, the aggregation models generated RRs around one, meaning no detectable estimated effect of PCV10, in the majority of the states (22 states with the quarter/state model, 23 states with the semester/state model, 25 states with the year/state model, 22 states with the month/region model, and 22 states with the month/nation model) (Figure 1, eFigure 2; http://links.lww.com/EDE/B785, and eFigure 3; http://links.lww.com/EDE/B785). The aggregation of controls improved the model fit (i.e., DIC values of the aggregation models became smaller than that of the reference model) in all 25 states, although the DIC evidence ratio and differences in the model likelihood were small in some states (Table and eTable 3; http://links.lww.com/EDE/B785). The aggregation model with the smallest value of DIC (referred as the “best” model for each state in Figure 2) adjusted for the long-term underlying trends and generated RRs closer to the null in some of the aforementioned 10 states where the reference model generated questionable estimates of RRs (e.g., Tocantins, Alagoas, Goiás, and Distrito Federal).

**TABLE 1.** Deviance Information Criterion Evidence Ratios Comparing the Reference Model to the Best Models of Approaches 1 and 2

State	Rate Ratio by the Reference Model (95% CrI)	Average Population	Approach 1 (Simple Aggregation)		Approach 2 (DLM)	
			DIC	Best Model	DIC	Best Model
Acre	1.35 (1.03–1.68)	4,671	2.1	Quarter/state	0.9	Spatial DLM
Rondônia	1.32 (1.06–1.64)	7,965	8	Year/state	6.4	Spatial DLM
Distrito Federal	1.2 (1.01–1.42)	8,273	2,631.4	Year/state	223.1	Temporal DLM
Tocantins	1.33 (1.12–1.59)	10,474	25.9	Quarter/state	11	Spatial DLM
Amazonas	1.14 (0.93–1.4)	18,763	180.6	Year/state	2.1	Temporal DLM
Mato Grosso	1.29 (1.07–1.45)	18,808	1.5	Month/regional	1	Spatial DLM
Sergipe	1.46 (1.19–1.91)	21,729	1.2	Semester/state	0.8	Spatial DLM
Mato Grosso do Sul	1.35 (1.16–1.58)	23,016	1.6	Month/national	1.8	Temporal DLM
Alagoas	1.39 (1.15–1.62)	29,414	12	Semester/state	276	Spatial DLM
Piauí	1.03 (0.87–1.23)	31,741	5.7	Year/state	1.3	Temporal DLM
Espírito Santo	1.18 (0.93–1.44)	32,103	86.9	Semester/state	308.1	Temporal DLM
Rio Grande do Norte	0.99 (0.81–1.22)	44,067	1,343.6	Year/state	53.8	Spatial DLM
Pará	1.06 (0.83–1.31)	48,667	4.4	Semester/state	1.8	Temporal DLM
Goiás	1.52 (1.22–1.76)	49,414	15.2	Quarter/state	7.2	Spatial DLM
Paraíba	1.19 (0.98–1.41)	57,171	2	Month/national	1.1	Temporal DLM
Santa Catarina	0.99 (0.86–1.14)	57,614	22.5	Month/national	96.5	Spatial DLM
Maranhão	1.03 (0.76–1.35)	58,709	18.4	Quarter/state	3.6	Spatial DLM
Paraná	0.94 (0.8–1.1)	96,581	80.1	Month/national	10.7	Spatial DLM
Pernambuco	1.37 (1.15–1.64)	107,174	4.5	Semester/state	2.1	Temporal DLM
Ceará	0.99 (0.85–1.16)	112,506	6	Semester/state	2.2	Temporal DLM
Rio Grande do Sul	0.96 (0.83–1.11)	142,994	3.1	Year/state	8.7	Temporal DLM
Rio de Janeiro	1 (0.85–1.18)	163,895	6.3	Quarter/state	1.6	Temporal DLM
Bahia	0.94 (0.82–1.07)	181,109	1.5	Month/regional	1.2	Temporal DLM
Minas Gerais	0.88 (0.75–1.01)	216,353	1.1	Month/regional	1.9	Temporal DLM
São Paulo	0.99 (0.86–1.13)	350,428	1.1	Quarter/state	1.2	Temporal DLM

Best models were defined as the models with the smallest DIC values for each state.

The aggregation models were able to capture long-term underlying trends because they identified stronger relationships between the outcome and controls. For example, in Tocantins (the fourth smallest state in terms of population), an estimated coefficient for K00_99 (diseases of the digestive system) was 0.024 in the reference model, whereas it was 0.270 and 0.218 in the quarterly and semiannual model, respectively (eTable 4; http://links.lww.com/EDE/B785). The other mechanism that helped models to adjust for underlying trends was that the spatial aggregation models were able to use information on a larger number of control diseases, by borrowing data from neighboring states. For example, Alagoas had information on 11 control diseases available (eTable 5; http://links.lww.com/EDE/B785). By incorporating data from other states in the same region or from all states in the country, the spatial aggregation models were able to include 22 or 24 control diseases, respectively, in the regression. The spatial aggregation models selected more control diseases (i.e., relative importance weights were >0.50 in eTable 5; http://links.lww.com/EDE/B785), resulting in more reliable estimates of the RRs (Figure 2).

### Approach 2: Distributed Lag Models

The DLMs, which automatically selected an appropriate set of controls and their aggregation levels, also generated similar results by employing lagged information. Compared to the reference model, the DLMs had larger DIC evidence ratios (Table) and model likelihood (eTable 3; http://links.lww.com/EDE/B785) in 23 of 25 states, although differences were sometimes small. The DLMs were also able to capture the underlying trends by incorporating lagged information in some states, such as Alagoas, Goiás, and Distrito Federal, and estimated RRs moved toward the null (Figures 1E and H and 2). The temporal DLM resulted in reduced posterior uncertainty surrounding the RR estimates when compared to the other temporal aggregation methods. The spatial DLMs tend to outperform in smaller states, as they were able to use the information on more control diseases provided by surrounding states (Table and eTable 5; http://links.lww.com/EDE/B785). In larger states, the spatial lagged information was less important and temporal DLMs were preferred (Table).

## DISCUSSION

The synthetic control method is a powerful tool to disentangle changes caused by an intervention of interest from those caused by other unaffected time-varying factors. The model, however, can fail to adjust for trends when control disease time series are sparse. In Brazil, the reference model using the original, nonaggregated control time series suffered from this issue, and estimated that there was an increase in pneumonia hospitalizations in the postvaccine period especially in states with sparse data. Due to limited healthcare access and resources and small population size, low-income states were more likely to have sparse data, making it challenging for the synthetic control model to adjust for underlying trends and obtain robust estimates of the impact of PCV in these states. To overcome this issue, we proposed the use of aggregated control time series. Aggregation of controls enabled the models to capture relationships between the outcome and controls more effectively and to use information on more control diseases by borrowing data from neighboring geographic areas. As a result, the models successfully adjusted for underlying trends and resulted in more robust estimates of the impact of PCV10 in some of the states where the reference model failed to adjust for underlying trends. In other states, however, the use of aggregated controls did not improve the model performance. One of the reasons is that their population size was too small and control time series were still very noisy/sparse even after aggregation. It suggests that aggregation of control time series is not a perfect solution that can be universally applied to all states; it is rather one of many ways that researchers can try when this issue arises.

A question then becomes how to choose an appropriate aggregation level. The DLMs are useful tools that automate this process, including the selection of controls and their aggregation levels. All steps are carried out in one seamless procedure. Additionally, the DLM can essentially collapse to the standard synthetic control model in the case that lagged information is not helpful. One disadvantage is that the temporal DLM needs to remove lag periods at the beginning of the time series data, which might become an issue when only a short period of prevaccine data is available.

We used DIC to compare the performances of the standard synthetic control (SC) model, aggregation SC models, and DLMs. Although the aggregation models and DLMs had smaller DIC values than the reference model in all 25 states and in 23 states, respectively, the differences in DIC values were sometimes negligible. Focusing solely on model fit in the prevaccine period as DIC does may not tell the entire story in terms of predicting accurate counterfactuals. Thus, as a supplementary analysis, we aimed to compare predictive performance of the models using a mean absolute error of the predictions for all-cause pneumonia hospitalizations averaged across the first 12 months of the postvaccine period (eTable 6; http://links.lww.com/EDE/B785). We assumed that PCV had not yet impacted hospitalizations during these months, as the elderly were not routinely immunized by PCV and the impact of herd immunity takes a longer time to appear. There was no consistent “winner” among these models; however, in 10 states where the reference model failed to adjust for long-term increasing trends during the evaluation period (i.e., RRs > 1 in the main analysis), one of the aggregation models or DLMs had the smallest mean absolute error, supporting our main conclusion that these approaches yielded more reliable estimates of the impact of PCV in states with sparse data. Generally, this approach may be problematic for applications with shorter time series lengths, as the focus is on the longer-term predictive ability of the methods which requires more prevaccine data for validation. Therefore, alternative approaches may be needed to formally compare the explanatory and predictive performance of these types of models.

In conclusion, when the synthetic control model fails to select appropriate controls because of the noise in control time series, we propose that users try aggregating control time series, either temporally or spatially. It helps the synthetic control model to select appropriate control diseases and can lead to more robust estimates of vaccine impact. The DLMs can automatically select the appropriate set of controls and their aggregation levels.

## Supplementary Material


