# Supplementary material for: Incorporating Information on Control Diseases Across Space and Time to Improve Estimation of the Population-level Impact of Vaccines
Source: Epidemiology. 2021 Mar 12;32(3):360–7. doi: 10.1097/EDE.0000000000001341 (PMC8011507; doi:10.1097/EDE.0000000000001341)
Supplement: Supplementary file 1 [file ede-32-360-s001.pdf]

eTable 1. List of control diseases included in the synthetic control model.

Grouping scheme	ICD-10		Exclusions
ICD-10 chapters			
	C00_D48	Neoplasms	
	D50_89	Diseases of blood and blood-forming organs and certain disorders involving the immune mechanism	
	E00_99	Endocrine, nutritional, metabolic disorders	
	G00_99_SY	Diseases of the nervous system	G00_04
	H00_99_SY	Diseases of the ear and mastoid process	H10, H65, H66
	I00_99	Diseases of the circulatory system	
	K00_99	Diseases of the digestive system	
	L00_99	Diseases of the skin	
	M00_99	Diseases of the musculoskeletal system	
	N00_99	Diseases of the genitourinary system	
	P00_99	Perinatal diseases	
	Q00_99	Congenital malformations, deformations and chromosomal abnormalities	
	S00_T99	Injury, poisoning and consequences of external causes	
	Z00_99	Factors influencing health status and contact with health workers	
Other grouped outcomes			
	a10_b99_nopneumo	Certain infectious and parasitic diseases, except intestinal	A40.3, B95
	E10_14	Diabetes	
	E40_46	Malnutrition	
	I60_64	Stroke	
	J20_22	Bronchitis, bronchiolitis and unspecified acute lower respiratory infection	
	ach_noj	All nonrespiratory hospitalizations	J00_99, F and O chapters
Specific outcomes			
	A41	Other septicemia	
	K35	Appendicitis	
	K80	Cholelithiasis	
	N39	Urinary tract infection	

Abbreviation: ICD, International Statistical Classification of Diseases and Related Health Problems.

eTable 2. Temporal and spatial aggregation levels for control time series used in synthetic control models in Approach 1.

**Model**	**Aggregation level for control time series**	
	**Time scale**	**Spatial scale**
Reference model	Month	State
Temporal aggregation 1	Quarter	State
Temporal aggregation 2	Semester	State
Temporal aggregation 3	Year	State
Spatial aggregation 1	Month	Region
Spatial aggregation 2	Month	Nation

eTable 3. DIC model likelihood comparing the models of Approach 1 and 2.

	Approach 1 (Simple aggregation)						Approach 2 (DLM)	
State	Month (Reference)	Quarter	Semester	Year	Region	Nation	Temporal DLM	Spatial DLM
Rondônia*	0.13	0.23	0.78	1.00	0.05	0.17	0.16	0.80
Acre*	0.47	1.00	0.50	0.57	0.51	0.43	0.39	0.44
Amazonas	0.01	0.01	0.78	1.00	0.01	0.01	0.01	0.01
Pará	0.22	0.50	1.00	0.75	0.17	0.59	0.40	0.36
Tocantins*	0.04	1.00	0.73	0.27	0.13	0.26	0.04	0.43
Maranhão	0.05	1.00	0.30	0.27	0.42	0.29	0.14	0.19
Piauí	0.18	0.63	0.92	1.00	0.19	0.86	0.22	0.17
Ceará	0.17	0.48	1.00	0.97	0.55	0.57	0.37	0.35
Rio Grande do Norte	0.00	0.01	0.03	1.00	0.08	0.03	0.00	0.04
Paraíba	0.50	0.25	0.41	0.66	0.69	1.00	0.53	0.51
Pernambuco*	0.22	0.47	1.00	0.07	0.36	0.10	0.47	0.37
Alagoas*	0.00	0.01	0.04	0.01	0.01	0.01	0.00	1.00
Sergipe*	0.85	0.76	1.00	0.67	0.68	0.60	0.74	0.70
Bahia	0.65	0.58	0.78	0.29	1.00	0.38	0.75	0.55
Minas Gerais	0.54	0.40	0.31	0.20	0.58	0.17	1.00	0.66
Espírito Santo	0.00	0.19	0.28	0.05	0.03	0.05	1.00	0.01
Rio de Janeiro	0.16	1.00	0.64	0.33	0.32	0.36	0.25	0.16
São Paulo	0.82	0.91	0.89	0.57	0.82	0.64	1.00	0.73
Paraná	0.01	0.05	0.21	0.25	0.03	1.00	0.10	0.13
Santa Catarina	0.01	0.14	0.05	0.23	0.02	0.23	0.23	1.00
Rio Grande do Sul	0.12	0.14	0.19	0.36	0.17	0.28	1.00	0.28
Mato Grosso do Sul*	0.56	0.66	0.40	0.35	0.45	0.88	1.00	0.90
Mato Grosso*	0.67	0.58	0.81	0.68	1.00	0.91	0.65	0.66
Goiás*	0.07	1.00	0.91	0.29	0.16	0.93	0.13	0.48
Distrito Federal*	0.00	0.15	0.57	1.00	0.01	0.16	0.08	0.02

* The reference model generated rate ratios with 95% credible interval lower bounds >1, suggesting that pneumonia hospitalizations increased after vaccine introduction, in these ten states.

Models with larger DIC likelihood are highlighted in darker red.

Abbreviations: DIC, deviance information criterion; DLM, distributed lag model.

eTable 4. Estimated coefficients (beta) for the control diseases in the temporal aggregation models (Tocantins, Brazil).

**Control diseases**	**Month (reference)**	**Quarter**	**Semester**	**Year**	**Temporal DLM**
a10_b99_nopneumo	0.001	0.000	0.001	0.031	0.006
Non-respiratory hospitalizations	**0.127**	0.005	0.018	0.017	**0.097**
C00_D48	0.000	0.037	0.067	0.026	0.02
E00_99	**0.014**	0.000	-0.003	-0.005	0.03
E10_14	-0.013	**-0.068**	**-0.185**	**-0.097**	-0.039
I00_99	0.000	-0.011	-0.030	-0.007	0.002
I60_64	0.005	**0.059**	**0.159**	**0.107**	0.015
K00_99	**0.024**	**0.270**	**0.218**	0.021	**0.055**
N00_99	0.001	-0.001	0.022	0.050	0.000
S00_T99	-0.001	0.003	0.017	0.015	-0.009

Numbers are in bold if their relative importance weights are >0.50. The definition of ICD-10 codes can be found in Table S1.

Abbreviations: DLM, distributed lag model; ICD, International Statistical Classification of Diseases and Related Health Problems.

eTable 5. Number of controls selected by the Bayesian variable selection and the number of controls available (in parenthesis) for adults aged ≥80 years in each state in Brazil.

State	Approach 1 (Simple aggregation)						Approach 2 (DLM)	
	Month (reference)	Quarter	Semester	Year	Region	Nation	Temporal DLM	Spatial DLM
Rondônia*	1 (9)	2 (9)	2 (9)	2 (9)	3 (19)	5 (24)	1 (9)	5 (24)
Acre*	0 (3)	0 (3)	0 (3)	0 (3)	2 (19)	0 (24)	0 (3)	4 (24)
Amazonas	3 (12)	2 (12)	2 (12)	4 (12)	4 (19)	5 (24)	2 (12)	4 (24)
Pará	2 (17)	5 (17)	3 (17)	3 (17)	4 (19)	5 (24)	4 (17)	3 (24)
Tocantins*	3 (10)	3 (10)	3 (10)	2 (10)	5 (19)	6 (24)	2 (10)	6 (24)
Maranhão	3 (15)	5 (15)	2 (15)	1 (15)	3 (22)	3 (24)	3 (15)	3 (24)
Piauí	2 (12)	4 (12)	2 (12)	3 (12)	7 (22)	4 (24)	1 (12)	6 (24)
Ceará	3 (19)	3 (19)	3 (19)	3 (19)	6 (22)	6 (24)	6 (19)	6 (24)
Rio Grande do Norte	3 (13)	3 (13)	3 (13)	3 (13)	4 (22)	4 (24)	3 (13)	6 (24)
Paraíba	3 (17)	5 (17)	3 (17)	3 (17)	4 (22)	5 (24)	5 (17)	8 (24)
Pernambuco*	6 (18)	4 (18)	7 (18)	3 (18)	6 (22)	5 (24)	5 (18)	5 (24)
Alagoas*	2 (11)	2 (11)	1 (11)	2 (11)	7 (22)	7 (24)	2 (11)	7 (24)
Sergipe*	2 (9)	2 (9)	1 (9)	0 (9)	2 (22)	1 (24)	2 (9)	5 (24)
Bahia	4 (20)	4 (20)	4 (20)	2 (20)	3 (22)	4 (24)	4 (20)	7 (24)
Minas Gerais	8 (20)	5 (20)	6 (20)	5 (20)	3 (22)	7 (24)	5 (20)	6 (24)
Espírito Santo	4 (18)	3 (18)	3 (18)	4 (18)	3 (22)	4 (24)	3 (18)	3 (24)
Rio de Janeiro	4 (20)	5 (20)	6 (20)	5 (20)	8 (22)	5 (24)	5 (20)	6 (24)
São Paulo	4 (21)	4 (21)	7 (21)	3 (21)	3 (22)	8 (24)	6 (21)	6 (24)
Paraná	7 (20)	4 (20)	5 (20)	5 (20)	7 (20)	8 (24)	5 (20)	5 (24)
Santa Catarina	5 (17)	4 (17)	4 (17)	5 (17)	6 (20)	5 (24)	6 (17)	4 (24)
Rio Grande do Sul	6 (20)	6 (20)	4 (20)	2 (20)	4 (20)	7 (24)	2 (20)	5 (24)
Mato Grosso do Sul*	2 (13)	3 (13)	3 (13)	2 (13)	6 (20)	6 (24)	3 (13)	5 (24)
Mato Grosso*	0 (10)	0 (10)	0 (10)	1 (10)	4 (20)	3 (24)	0 (10)	3 (24)
Goiás*	3 (17)	3 (17)	2 (17)	5 (17)	5 (20)	7 (24)	5 (17)	4 (24)
Distrito Federal*	3 (11)	2 (11)	3 (11)	2 (11)	4 (20)	5 (24)	2 (11)	4 (24)

* The reference model generated rate ratios with 95% credible interval lower bounds >1, suggesting that pneumonia hospitalizations increased after vaccine introduction, in these ten states.

Abbreviation: DLM, distributed lag model.

eTable 6. Out of sample evaluation of model performance.

Model	% Smallest MAE	Average change in MAE compared to the reference model
**Reference (month/state)**	4%	Reference
**Quarter/state**	16%	2.4%
**Semester/state**	12%	3.8%
**Year/state**	0%	1.1%
**Month/Region**	24%	-5.7%
**Month/Nation**	24%	-8.8%
**Spatial DLM**	4%	-4.3%
**Temporal DLM**	16%	-2.1%

Models were fit to data from the pre-vaccine period, and predictions were compared to the observed data from the first 12 months of post-vaccine data (March 2010-February 2011). The middle column indicates the proportion of states for which the indicated model had the lowest mean absolute error (MAE). The last column shows the average change in MAE compared to the reference model across all states. Negative numbers indicate an improvement in MAE compared to the reference.

Abbreviations: MAE, mean absolute error; MSE, mean squared error; DLM, distributed lag model.

eFigure 1. An example of the control disease time series (S00-T99: injury, poisoning and consequences of external causes) aggregated by month, quarter, semester, and year among adults aged ≥80 years in Paraná, Brazil.

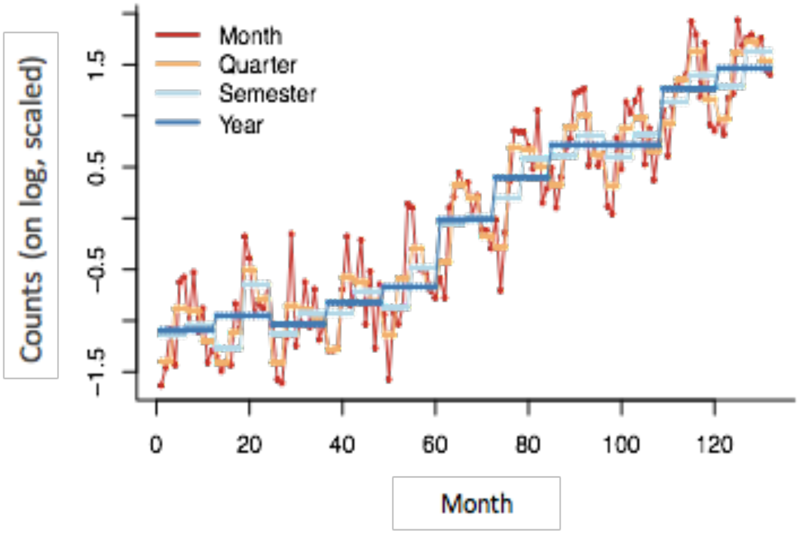

The graph shows time series for S00-T99 (injury, poisoning and consequences of external causes) for adults aged ≥80 years in Paraná, aggregated at different temporal scales (month, quarter, semester, or year). The counts are scaled and transformed to a log scale.

eFigure 2. Rate ratios for adults aged ≥80 years in 25 states in Brazil, estimated by the synthetic control models using control time series aggregated by time and the temporal lag model.

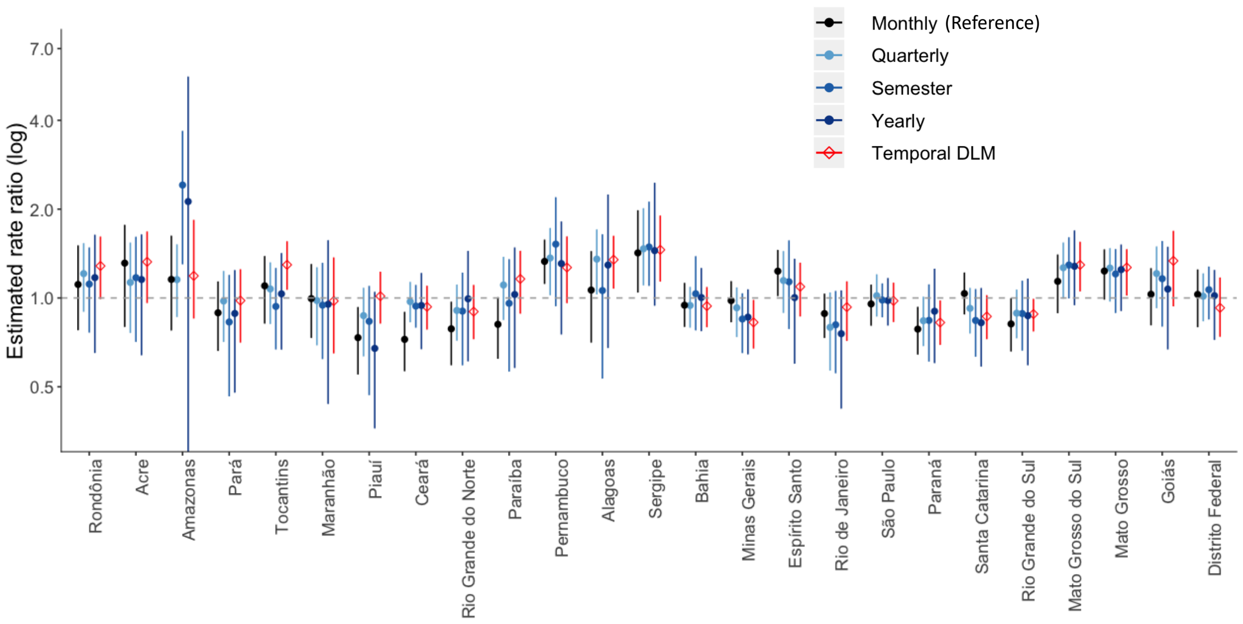

Rate ratios were the cumulative number of observed all-cause pneumonia hospitalizations (ICD-10 code: J12-18) divided by the cumulative number of counterfactual pneumonia hospitalizations in the evaluation period (March 2011-December 2015). Dots and bars represent posterior medians and 95% highest density credible intervals.

Abbreviations: DLM, distributed lag model; ICD, International Statistical Classification of Diseases and Related Health Problems.

eFigure 3. Rate ratios for adults aged ≥80 years in 25 states in Brazil, estimated by the synthetic control models using control time series aggregated by space and the spatial lag model.

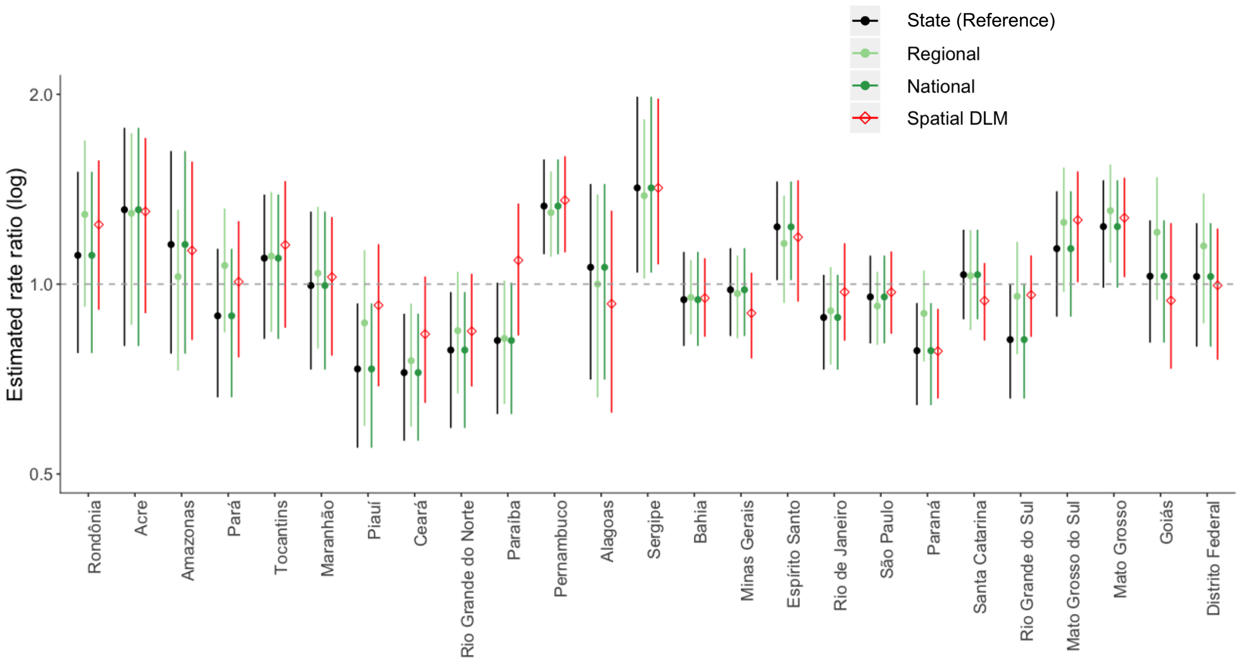

Rate ratios were the cumulative number of observed all-cause pneumonia hospitalizations (ICD-10 code: J12-18) divided by the cumulative number of counterfactual pneumonia hospitalizations in the evaluation period (March 2011-December 2015). Dots and bars represent posterior medians and 95% highest density credible intervals.

Abbreviations: DLM, distributed lag model; ICD, International Statistical Classification of Diseases and Related Health Problems.
